# Our Defences against Smallpox

**Published:** 1919-04-05

**Authors:** 


### OUR DEFENCES AGAINST SMALLPOX.

In the recent Milroy Lectures on smallpox and vaccination given by John C. McVail, M.D., LL.D., it is pointed out that this country has for some years shown a decreasing number of recently vaccinated people—in fact, it is now some time since an organised crusade for vaccination of either adults or children has occurred. This is partly due to the remarkable freedom of Great Britain from smallpox in the last seven or eight years, and partly to the greatly reduced virulence observed in those small outbreaks which have occurred both here and in America, allowing ready limitation of spread by the usual methods of isolation, contact observation, etc.

The lecturer gives conclusive proof of the protection which vaccination has provided in a series of concrete examples, such as the threatened outbreak in Glasgow in 1901-2, at the time of that city's great international exhibition. From beginning to end infections were limited to that section of the population *not* recently vaccinated. Among those who were inoculated not a single case occurred, and fully a year later, when another epidemic broke out, only one case appeared among them. At the same time he explains that smallpox as now seen in Western Europe, even when occurring in unvaccinated people, is a relatively mild attenuated form. The malignant and formerly common variety is still prevalent in the East, and although our present general measures are able to prevent the spread and make the disease almost unknown among an only sparsely vaccinated civil population in England, yet we must remember that should the malignant variety reappear there is considerable doubt as to the absolute efficiency of our protection.

At the moment all lines of flow from the Continent not only of people, but of disease, are under such strict surveillance that this precaution alone provides a strong shield, but later there will be far freer and less closely watched intercommunication, and with it the possibility of disease entry to this country. It is known that the thousands of Russians detained by Germany as prisoners of war brought the old malignant form of smallpox into the then "Central Empires," and the severity of the outbreaks was probably greater than enemy reports would have us believe. Cases are still appearing there, and it is against these and their contacts that all neighbouring European countries must beware.

One cannot but feel that some active measures, Governmental or professional, should be taken to raise the proportion of people vaccinated. There is no need to wait for the outbreak; immunity will continue for a reasonable length of time, and to us it seems that in the present days this matter calls for attention quite as strongly as many more-talked-of matters.

